# The effect of left atrial remodeling after cryoballoon ablation and radiofrequency ablation for paroxysmal atrial fibrillation

**DOI:** 10.1002/clc.23507

**Published:** 2020-11-18

**Authors:** Xule Wang, Beibei Song, Chunguang Qiu, Zhanying Han, Xi Wang, Wenjie Lu, Xiaojie Chen, Yingwei Chen, Liang Pan, Guoju Sun, Xiaofei Qin, Ran Li

**Affiliations:** ^1^ Department of Cardiology The First Affiliated Hospital of Zhengzhou University Zhengzhou PR China

**Keywords:** atrial remodeling, cryoballoon ablation, paroxysmal atrial fibrillation, radiofrequency ablation

## Abstract

**Background:**

Cryoballoon ablation (CBA) and radiofrequency ablation (RFA) are the most common procedures used to treat refractory atrial fibrillation (AF) and are performed through pulmonary vein isolation (PVI). Studies have shown that CBA can approximately match the therapeutic effects of RFA against AF. However, few studies have investigated the difference between CBA and RFA of the effects on left atrial remodeling for paroxysmal AF.

**Objective:**

Atrial remodeling is considered pivotal to the occurrence and development of AF, therefore we sought to assess the influence of atrial remodeling in patients with paroxysmal AF after CBA and RFA in this study.

**Methods:**

In this nonrandomized retrospective observational study, we enrolled 328 consecutive patients who underwent CBA or RFA for refractory paroxysmal AF in May 2014 to May 2017 in our hospital. After propensity score matching, 96 patients were included in the CBA group, and 96 were included in the RFA group. Patients were asked to undergo a 12‐lead electrocardiogram, a 24‐h Holter monitor, and an echocardiogram and to provide their clinical history and symptoms at 6 months and 1, 2, and 3 years postprocedurally. Electrical remodeling of the left atrium was assessed by P wave dispersion (Pdis); structural remodeling was assessed by the left atrium diameter (LAD) and left atrial volume index (LAVI) during scheduled visits.

**Results:**

As of January 2020, compared with baseline, at 1 year, 2 years, and 3 years after ablation, the average changes in Pdis (∆Pdis), LAD (∆LAD), and LAVI (∆LAVI) were significant in both the CBA and RFA groups. Six months after ablation, ∆Pdis, ∆LAD, and ∆LAVI were greater in the CBA group than in the RFA group. There was no significant difference between the two groups in AF/flutter recurrence, but the AF/flutter‐free survival time of CBA group may be longer than RFA group after 2 years after ablation. A higher ∆Pdis, ∆LAD, or ∆LAVI at 1 year after ablation may increase AF/flutter‐free survival.

**Conclusions:**

Although CBA and RFA are both effective in left atrial electrical and structural reverse‐remodeling in paroxysmal AF, CBA may outperform RFA for both purposes 6 months after ablation. However, during long‐term follow‐up, there was no significant intergroup difference.

## 1 INTRODUCTION

Previous studies have shown that the occurrence and development of AF are closely related to atrial remodeling, which includes neural remodeling, electrical remodeling and structural remodeling.^1^, ^2^, ^3^ In recent years, as catheter ablation based on pulmonary vein isolation (PVI) has become accepted as the cornerstone of all AF ablation procedures to maintain sinus rhythm, studies have been performed to observe the clinical outcomes of both cryoballoon ablation (CBA) and radiofrequency ablation (RFA). Evaluations of the differences in the safety and effectiveness of these two procedures have shown that CBA can achieve therapeutic effects similar to those of RFA in the treatment of paroxysmal AF.^4^, ^5^, ^6^, ^7^

Several studies have explored the effects of catheter ablation on left atrial size^8^, ^9^, ^10^, ^11^ and electrical conduction^12^, ^13^, ^14^, ^15^ in patients with AF, but no one has compared the influence of atrial remodeling caused by CBA and RFA in paroxysmal AF.

## 2 METHODS

### 2.1 Patients

We searched for patients who underwent CBA or RFA for paroxysmal AF in May 2014 to January 2017 at the First Affiliated Hospital of Zhengzhou University. Patients were selected according to the inclusion and exclusion criteria. All patients were required to have complete medical records, including age, sex, height, weight, and history of AF and comorbidities, as well as a preprocedural transesophageal echocardiogram to verify the absence of intracardiac thrombus and a computed tomography angiography (CTA) imaging to review the anatomy of the left atrium and PVs before the procedure. The management of oral anticoagulation during the perioperative period was determined by the physician according to the CHA2DS2‐VASc score. We enrolled patients according to the following criteria: patients aged 18 to 75 years with paroxysmal AF who had undergone RFA or CBA. Exclusion criteria included a previous history of catheter ablation for AF, complicated with valvular heart disease, hepatic and renal insufficiency, hyperthyroidism, cardiomyopathy, and implanted pacemakers or defibrillators.

### 2.2 Follow‐up

Patients were followed up by WeChat platform and outpatient service at 6 months and 1, 2, and 3 years after ablation, assessed for clinical history and symptoms, and asked for a 12‐lead electrocardiogram, a 24‐h Holter monitor, and an echocardiogram. Patients who presented with symptoms such as palpitations and chest tightness were asked to undergo a 12‐lead electrocardiogram or a 24‐h Holter monitor immediately and to transmit the ECGs to us. Atrial fibrillation (AF)/flutter‐free survival was defined as the absence of AF and atrial flutter recurrence ≥30 seconds following a 3‐month blanking period. AF/flutter recurrence was defined as documented recurrence of AF or atrial flutter (lasting more than 30 s) after the 90‐day blanking period or repeat ablation.^6^ Electrical remodeling of the left atrium was assessed by P wave dispersion (Pdis)^16^, ^17^, ^18^ as measured by a 12‐lead electrocardiogram, while structural remodeling was assessed by the left atrium diameter (LAD) and left atrial volume index (LAVI) as measured by echocardiogram during scheduled visits.^19^, ^20^, ^21^ Pdis was defined as the difference between the maximum and minimum P‐wave durations recorded by the 12 ECG leads.^17^, ^22^ Minimum three consecutive beats were measured in all simultaneous 12 leads to calculate P‐wave duration. LAD and left atrial volume (LAV) were measured using real‐time three‐dimensional echocardiography technology, then, the LAV was converted to LAVI by dividing by body surface area. Evaluation of each measurement was repeated three times to obtain the mean value for analysis. The postprocedural Pdis, LAD, and LAVI of each patient were subtracted from the Pdis, LAD, and LAVI at baseline to obtain the changes in Pdis, LAD, and LAVI of each patient, and then we could obtain the mean values of the changes in Pdis, LAD, and LAVI, that is, ΔPdis, ΔLAD, and ΔLAVI, in the CBA group and the RFA group. Follow‐up was ended once the patient underwent a repeat ablation after AF/flutter recurrence.

### 2.3 Ablation procedure

During CBA, a second‐generation cryoablation catheter and *Achieve* electrodes were advanced to the orifice of the PV. Once or twice cryoballoon freezes (each with a duration of 180 seconds) was performed to achieve PV isolation in each pulmonary vein. For RFA, after a three‐dimensional reconstruction of the left atrium, saline‐irrigated electrode was used to perform pulmonary vein antrum isolation (PAVI) until complete PVI is obtained.

### 2.4 Statistical analysis

Analyses were conducted with IBM SPSS Statistics, version 23. Given the differences in baseline characteristics between the CBA group and RFA group, we matched the CBA group to the RFA group using propensity score matching at a 1:1 ratio.

Continuous data are reported as the mean and standard deviation (SD), and categorical data are expressed as percentages. A *t*‐test was used to compare the differences in Pdis, LAD, and LAVI obtained at baseline and postprocedurally; another *t*‐test was used to compare the differences in the average changes in Pdis, LAD, and LAVI between the CBA group and the RFA group; a chi‐square test was used to evaluate the difference in the postoperative AF/flutter recurrence rate; and the Kaplan‐Meier method was used to compare the AF/flutter‐free survival times between the two groups. Logistic regression was used to explore the association between changes in indices (ΔPdis, ΔLAD, and ΔLAVI) and AF/flutter‐free survival. Values of *P* < .05 were considered significant.

## 3 RESULTS

A total of 328 consecutive patients were enrolled in this study. Of these patients, 223 (68.0%) underwent RFA, while 105 (32.0%) underwent CBA. The RFA group was more likely than the CBA group to have coronary artery disease or to have used an antiarrhythmic drug before matching. Five patients in the CBA group and 22 patients in the RFA group underwent repeat ablation after recurrence. After propensity score matching, 96 patients in the CBA group and 96 patients in the RFA group met our study inclusion criteria. The distributions of the propensity scores were comparable and all baseline characteristics were similar between the two groups (shown in Table 1). Of 192 patients, 50 (52.1%) in RFA group and 46 (47.9%) in CBA group received Antiarrhythmic drug after cryoablation, and stopped by the end of the blanking period.

**TABLE 1:** Characteristics of the patients at baseline

Characteristic	Before PSM			After PSM		
	RFA (N = 223)	CBA (N = 105)	*P* value	RFA (N = 96)	CBA (N = 96)	*P* value
Age (yr)	57.74 ± 10.17	57.12 ± 10.12	.50	57.59 ± 11.23	57.67 ± 10.00	.62
Male sex—no. (%)	143 (54.3)	55 (52.4)	.63	48 (50.0)	47 (49.0)	.69
BMI	25.83 ± 2.77	25.88 ± 2.86	.79	25.75 ± 2.99	25.82 ± 2.78	.80
Months since first AF diagnosis	30.83 ± 38.53	30.75 ± 65.45	.91	32.81 ± 39.97	31.33 ± 71.70	.88
LAD (mm)	37.25 ± 6.51	37.12 ± 5.80	.91	37.25 ± 6.51	37.11 ± 6.04	.90
Pdis (ms)	44.17 ± 11.90	43.64 ± 11.57	.83	44.16 ± 13.32	44.24 ± 11.76	.97
LAVI (mL/m^2^)	39.44 ± 8.18	39.63 ± 8.50	.85	39.32 ± 7.04	39.52 ± 7.58	.88
*CHA2DS2‐VASc score*						
Distribution—no. (%)						
0	77 (34.5)	28 (26.7)	.15	32 (33.3)	28 (29.2)	.53
1	60 (26.9)	41 (39.0)	.03	28 (29.2)	37 (38.5)	.17
2	42 (18.8)	19 (18.1)	.87	18 (18.8)	18 (18.8)	1.00
3	33 (14.8)	15 (14.3)	.90	13 (13.5)	11 (11.5)	.66
4	9 (4.0)	2 (1.9)	.51	5 (5.2)	2 (2.1)	.44
5	2 (0.9)	0 (0)	1.00	0 (0)	0 (0)	1.00
6	0	0		0	0	
NYHA classification—no. (%)						
No heart failure	14 599 (65.0)	8537 (81.0)	.03	44 (45.8)	36 (37.5)	.24
Class I	4453 (19.7)	1126 (10.5)	.04	22 (22.9)	26 (27.1)	.51
Class II	2058 (9.0)	8438 (7.6)	.68	27 (28.1)	32 (33.3)	.43
Class III	1513 (6.7)	42 4 (3.8)	.29	3 (2.1)	2 (3.1)	1.00
Medical history—no. (%)						
Previous DCCV	0 (0)	1 (1.0)	.14	0 (0)	1 (1.0)	1.00
Previous stroke/TIA	22 (9.9)	13 (12.4)	.49	9 (9.4)	12 (12.5)	.49
Previous MI	5 (2.2)	1 (1.0)	.67	2 (2.1)	1 (1.0)	1.00
Previous PCI	13 (5.8)	3 (2.9)	.24	6 (6.3)	3 (3.1)	.50
Coronary artery disease	66 (29.6)	18 (17.1)	.02	23 (24.0)	18 (18.8)	.38
Hypertension—no. (%)	99 (44.4)	45 (42.9)	.79	44 (45.8)	45 (50.6)	.89
T2DM—no. (%)	50 (22.4)	17 (16.2)	.19	20 (20.8)	16 (16.7)	.46
Medication use—no. (%)						
Antiarrhythmic drug	148 (66.4)	50 (47.6)	<.001	50 (52.1)	46 (47.9)	.56
ACEI/ARB	95 (42.6)	46 (43.8)	.84	42 (43.8)	44 (45.8)	.77
Beta‐blocker	99 (44.4)	48 (45.7)	.82	47 (49.0)	46 (47.9)	.89
Anticoagulation drug	86 (38.6)	37 (35.2)	.56	33 (34.4)	36 (37.5)	.65

*Note*: The plus‐minus values are mean ± SD. Abbreviations: ACEI, angiotensin‐converting enzyme inhibitor; AF, atrial fibrillation; ARB, angiotensin receptor blocker; BMI, body‐mass index, defined as the weight in kilograms divided by the square of the height in meters; CBA, cryoballoon ablation; CHA2DS2‐VASc, (congestive heart failure, hypertension, age ≥75 years, diabetes mellitus, and stroke/transient ischemic attack/thromboembolism)‐(vascular disease, age range from 65 to 74, sex [female]), with hypertension defined as blood pressure higher than 140/90 mmHg; DCCV, direct current cardioversion; LAD, left atrial diameter; LAVI, left atrial volume index; MI, myocardial infarction; NYHA, New York Heart Association; PCI, percutaneous coronary intervention; Pdis, P wave dispersion; PSM, propensity score match; RFA, radiofrequency ablation; TIA, transient ischemic attack; T2DM, type 2 diabetes.

### 3.1 Structural and electrical remodeling

In the per‐protocol analysis, as shown in Table 2, compared with baseline, at 1, 2, and 3 years after ablation, the changes in Pdis, LAD, and LAVI were significant in both the CBA and RFA groups. At 6 months after ablation, the average changes in Pdis, LAD, and LAVI were larger in the CBA group than in the RFA group (shown in Table 3). However, there were no significant group differences in the other indices.

**TABLE 2:** Average P‐wave dispersion, left atrial diameter, and left atrial volume index

	Pdis (ms)		LAD (mm)		LAVI (mL/m^2^)	
	CBA group	RFA group	CBA group	RFA group	CBA group	RFA group
Before ablation	44.24 ± 11.76	44.16 ± 13.32	37.11 ± 6.04	37.25 ± 6.51	39.52 ± 7.58	39.32 ± 7.04
6 months after ablation	35.20 ± 9.24	39.83 ± 13.78	35.33 ± 4.84	37.46 ± 6.06	36.24 ± 7.54	38.06 ± 8.87
1 year after ablation	34.81 ± 13.47*	38.43 ± 15.70*	34.72 ± 5.93*	35.06 ± 5.93*	34.52 ± 9.62*	35.77 ± 8.38*
2 years after ablation	30.48 ± 10.84*	35.60 ± 14.17*	32.68 ± 4.59*	33.18 ± 5.68*	31.74 ± 8.76*	33.48 ± 7.75*
3 years after ablation	29.09 ± 13.40*	33.86 ± 15.54*	30.11 ± 3.66*	31.64 ± 4.64*	30.27 ± 12.08*	31.02 ± 10.18*

*Note*: CBA, cryoballoon ablation; LAD, left atrial diameter; LAVI, left atrial volume index; Pdis, P‐wave dispersion; RFA, radiofrequency ablation.

^*^ *P* < 0.05: the difference between measurements obtained at baseline and after the procedure was significant.

**TABLE 3:** Average changes in P‐wave dispersion, left atrial diameter, and left atrial volume index

	CBA group	RFA group
∆Pdis‐6 months (ms)	6.45 ± 8.31*	2.22 ± 7.63*
∆Pdis‐1 year (ms)	9.15 ± 11.10	5.54 ± 9.48
∆Pdis‐2 years (ms)	11.82 ± 12.97	8.00 ± 13.39
∆Pdis‐3 years (ms)	12.36 ± 14.14	8.25 ± 12.24
∆LAD‐6 months (mm)	1.76 ± 2.36*	0.56 ± 1.91*
∆LAD‐1 year (mm)	2.49 ± 3.28	1.85 ± 2.96
∆LAD‐2 years (mm)	3.67 ± 5.30	3.27 ± 2.90
∆LAD‐3 years (mm)	4.22 ± 3.53	4.03 ± 3.16
∆LAVI‐6 months (mL/m^2^)	3.56 ± 4.73*	1.23 ± 3.66*
∆LAVI‐1 year (mL/m^2^)	4.99 ± 5.47	3.55 ± 5.56
∆LAVI‐2 year (mL/m^2^)	7.68 ± 7.78	6.29 ± 6.65
∆LAVI‐3 year (mL/m^2^)	8.85 ± 11.09	8.14 ± 8.50

*Note*: LAD, left atrial diameter; ∆LAD, the mean value of the change in LAD; LAVI, left atrial volume index; ∆LAVI, the mean value of the change in LAVI; Pdis, P‐wave dispersion; ∆Pdis, the mean value of the change in Pdis.

* *P* < 0.05: the difference between the CBA group and the RFA group was significant.

### 3.2 Atrial fibrillation/flutter recurrence and atrial fibrillation/flutter‐free survival time

As of January 2020, AF/flutter recurrence had recurred in 33(34.4%) patients in the CBA group and 39(40.6%) in the RFA group, but there was no significant difference between the two groups (Chi‐square = 1.047, *P* = .592).

**FIGURE 1:**
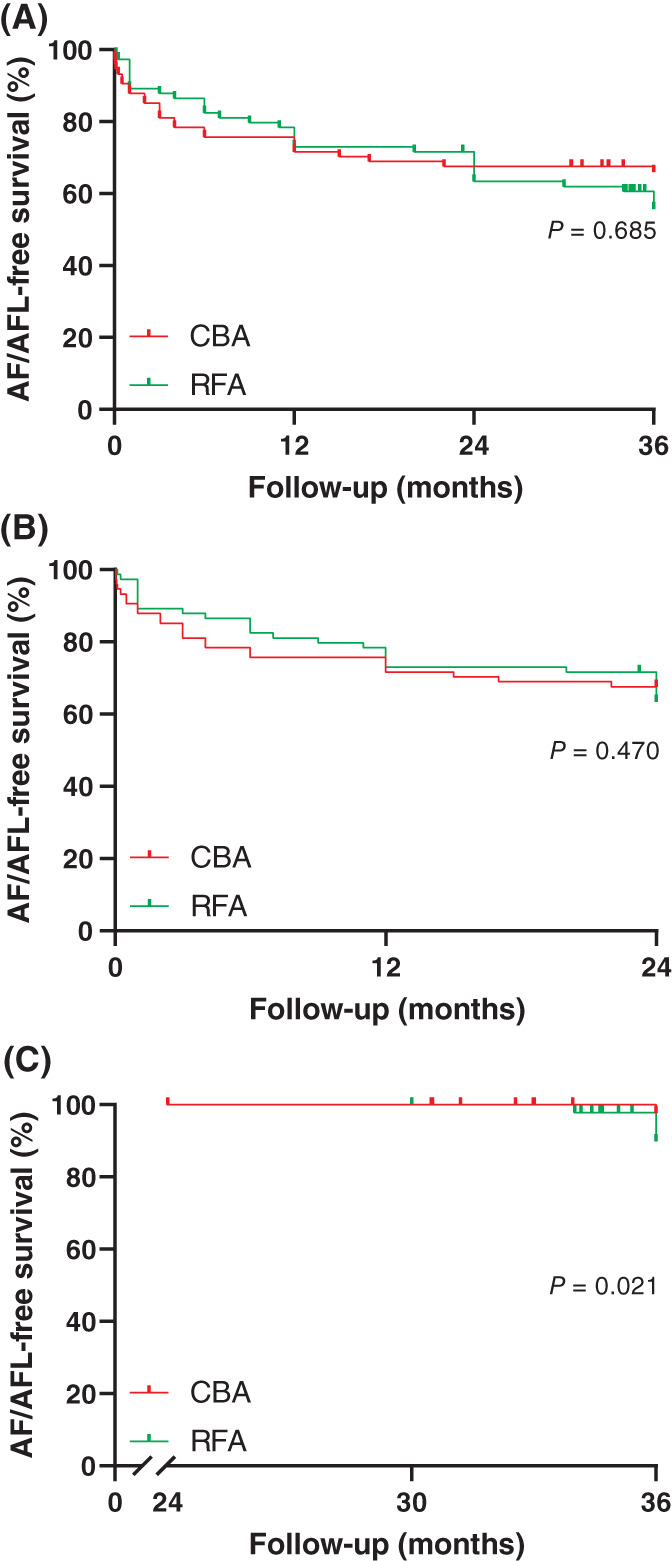
Survival plots of patients. A, Survival plots of all enrolled patients after propensity score matching. B, Survival plots of patients with an atrial fibrillation/flutter‐free survival time < 24 months after propensity score matching. C, Survival plots of patients with an atrial fibrillation/flutter‐free survival time ≥ 24 months after propensity score matching. CBA, cryoballoon ablation; RFA, radiofrequency ablation

The Kaplan‐Meier method was used to compare the AF/flutter‐free survival time between the two groups (shown in Figure 1). The mean AF/flutter‐free survival times of the two groups are shown in Table 4. The log rank method was used to evaluate the difference in AF/flutter‐free survival time, and there was no significant difference between the two groups (Chi‐square = 0.164, *P* = .685). Then, we compared the AF/flutter‐free survival times in cases with recurrence within 2 years and after 2 years between the CBA and RFA groups and found that the survival time of CBA group may be longer than RFA group after 2 years after ablation (shown in Figure 1 and Table 4).

**TABLE 4:** Comparison of mean atrial fibrillation/flutter‐free survival time between the CBA group and the RFA group

Patients	CBA group		RFA group			
	Mean atrial fibrillation/flutter‐free survival time	95% CI	Mean atrial fibrillation/flutter‐free survival time	95% CI	Chi‐square	*P* value
All enrolled	25.820	(22.347, 29.294)	26.127	(22.982, 29.273)	0.164	.685
Recurrence within 2 years	12.930	(10.056, 15.803)	11.447	(8.249, 14.645)	0.522	.470
Recurrence after 2 years	36.000	(36.000, 36.000)	34.129	(32.813, 35.445)	5.306	.021

*Note*: CBA, cryoballoon ablation; RFA, radiofrequency ablation.

### 3.3 Associations of ΔPdis, ΔLAD, and ΔLAVI with atrial fibrillation/flutter‐free survival

A logistic regression model analysis was performed, and we found that a higher ∆Pdis, ∆LAD, or ∆LAVI at 1 year after ablation increased AF/flutter‐free survival (OR 1.307, 95% CI 1.039‐1.605, *P* = .035; OR 1.301, 95% CI 1.089‐1.522, *P* < .001; OR 1.879, 95% CI 1.711‐1.963, *P* < .001, respectively).

## 4 DISCUSSION

### 4.1 Main findings

This is an observational clinical study aimed at exploring differences in atrial reverse‐remodeling in paroxysmal AF patients who underwent CBA or RFA. In this study, we found that Pdis, LAD, and LAVI significantly decreased in both groups during the follow‐up period. CBA may have performed better than RFA with regard for electrical and structural reverse‐remodeling at 6 months after ablation, but over the long‐term follow‐up period, there was no significant difference between the two groups. CBA may have a longer AF/flutter‐free survival time, and left atrial electrical and structural reverse‐remodeling may have a positive effect on AF/flutter‐free survival after ablation.

### 4.2 Structural and electrical remodeling

The mechanism of occurrence and maintenance of AF remains unclear, but evidence indicates that rapid focal activation originating from the pulmonary veins may play a pivotal role. However, catheter ablation for AF remains challenging, with a low success rate over long‐term follow‐up.^5^, ^19^ Previous studies have shown that RFA can reduce left atrium size and volume in patients without recurrence.^9^, ^10^ Similarly, Canpolat et al^12^ found that successful CBA improved electrical conduction in the left atrium and decreased the left atrium volume index. The results of our study are consistent with those presented in previous studies. The average changes in Pdis, LAD, and LAVI in patients with AF who underwent ablation clearly increased over time, indicating that ablation can indeed lead to reverse left atrial remodeling. A comparison between the two groups showed that ΔPdis, ΔLAD, and ΔLAVI were significantly larger in the CBA group than in the RFA group at 6 months after ablation. As Jun Kurse et al reported that the PVI lesions after CBA were wider and more continuous than RFA,^23^ we supposed the extension of ablation area produced by CBA may favor a higher entity of left atrial remodeling as compared to RFA. Wieczorek M.’s study^24^ compared RFA and CBA and found that CBA achieved a lower early pulmonary vein reconnection rate and may perform better in left atrial reverse‐remodeling in the early postablation period, similar to our finding that CBA may have longer AF/flutter‐free survival times. But we did not find a significant difference between the two groups over long‐term follow‐up, a larger sample size may be helpful to find an obvious difference.

### 4.3 Atrial fibrillation/flutter recurrence and atrial fibrillation/flutter‐free survival time

Compared with RFA, CBA has several advantages, such as better catheter stability, continuous and complete scar boundaries, and less damage to the endocardial surface and adjacent tissue and structures^25^; hence, CBA could potentially be noninferior to RFA with regard for safety and efficacy, as the FIRE AND ICE Trial showed over a 1.5‐year follow‐up duration.^6^ In terms of long‐term follow‐up, the STOP AF Trial obtained a ratio of 64.1% for freedom from AF and symptomatic atrial flutter/atrial tachycardia at 36 months after CBA in patients with paroxysmal AF.^5^ Another study reported a ratio of 41% freedom from atrial tachyarrhythmia recurrence after 31 ± 16 months of follow‐up following a single RFA procedure in nonparoxysmal AF.^26^ Our study compared the difference in the AF/flutter‐free survival rate between CBA and RFA during a three‐years‐follow‐up but did not show a significant difference. An important difference between our study and the aforementioned studies is that we evaluated clinical benefits according to the AF/flutter‐free survival time and sought to explore the relationship between reverse left atrial remodeling and clinical benefits. In our study, the AF/flutter recurrence rates and AF/flutter‐free survival times were not significantly different between the CBA and RFA groups, but the CBA group may have a longer survival time over 2 years after ablation.

### 4.4 Associations of ΔPdis, ΔLAD, and ΔLAVI with atrial fibrillation/flutter‐free survival

The DECAAF study found that progressive remodeling of the left atrium might be responsible for failure after ablation^27^; hence, we sought to explore whether AF/flutter‐free survival after ablation is associated with left atrial reverse‐remodeling. We found that ΔPdis, ΔLAD, and ΔLAVI at 1 year after ablation were positively correlated with AF/flutter‐free survival, indicating that left atrial electrical and structural reverse‐remodeling may have a positive effect on AF/flutter‐free survival after ablation.

## 5 LIMITATIONS

This study is a single‐center study with a small sample size, and large randomized studies are needed to confirm these data. Regrettably, we did not require long‐term continuous electrocardiographic monitoring after ablation, and the effects of ablation on the AF/flutter recurrence rate and AF/flutter‐free survival time may have been exaggerated. A more obvious difference may be found if a longer follow‐up time and a larger sample size are used to explore the difference in long‐term AF/flutter‐free survival time using these two methods.

## 6 CONCLUSIONS

Our results show that both CBA and RFA are effective in left atrial electrical and structural reverse‐remodeling in AF, and CBA may perform better in electrical and structural reverse‐remodeling at 6 months after ablation. However, over the long‐term follow‐up period, there was no significant difference between the two groups.

## ACKNOWLEGMENTS

This study was supported by the National Natural Science Foundation of China (81600271).

## CONFLICT OF INTEREST

The authors declare no potential conflict of interests.

## ETHICS STATEMENT

Informed written consent was signed by all patients. This study was approved by the ethics review committee of the First Affiliated Hospital of Zhengzhou University.

## Data Availability

Research data are not shared.

## References

[1] Iwasaki YK , Nishida K , Kato T , Nattel S . Atrial fibrillation pathophysiology: implications for management. Circulation. 2011;124(20):2264‐2274. 22083148 10.1161/CIRCULATIONAHA.111.019893

[2] Wang X , Huang C , Zhao Q , Tang Y , Yu S . Effect of renal sympathetic denervation on the inducibility of atrial fibrillation and structural remodeling in ambulatory canines with prolonged atrial pacing. Chin J Cardiac Pacing Electrophysiol. 2014;20(5):3043‐3064.

[3] Tondo C , Iacopino S , Pieragnoli P , et al. Pulmonary vein isolation cryoablation for patients with persistent and long‐standing persistent atrial fibrillation: clinical outcomes from the real‐world multicenter observational project. Heart Rhythm. 2018;15(3):363‐368. 29107190 10.1016/j.hrthm.2017.10.038

[4] Chen YH , Lu ZY , Xiang Y , et al. Cryoablation vs. radiofrequency ablation for treatment of paroxysmal atrial fibrillation: a systematic review and meta‐analysis. Europace. 2017;19(5):784‐794. 28065886 10.1093/europace/euw330

[5] Knight BP , Novak PG , Sangrigoli R , et al. Long‐term outcomes after ablation for paroxysmal atrial fibrillation using the second‐generation cryoballoon: final results from STOP AF post‐approval study. JACC Clin Electrophysiol. 2019;5(3):306‐314. 30898232 10.1016/j.jacep.2018.11.006

[6] Kuck KH , Brugada J , Furnkranz A , et al. Cryoballoon or radiofrequency ablation for paroxysmal atrial fibrillation(fire and ice trail). N Engl J Med. 2016;374(23):2235‐2245. 27042964 10.1056/NEJMoa1602014

[7] Murray MI , Arnold A , Younis M , Varghese S , Zeiher AM . Cryoballoon versus radiofrequency ablation for paroxysmal atrial fibrillation: a meta‐analysis of randomized controlled trials. Clin Res Cardiol. 2018;107(8):658‐669. 29564527 10.1007/s00392-018-1232-4

[8] Oka T , Inoue K , Tanaka K , et al. Left atrial reverse Remodeling after catheter ablation of nonparoxysmal atrial fibrillation in patients with heart failure with reduced ejection fraction. Am J Cardiol. 2018;122(1):89‐96. 29703439 10.1016/j.amjcard.2018.03.026

[9] Jeevanantham V , Ntim W , Navaneethan SD , et al. Meta‐analysis of the effect of radiofrequency catheter ablation on left atrial size, volumes and function in patients with atrial fibrillation. Am J Cardiol. 2010;105(9):1317‐1326. 20403486 10.1016/j.amjcard.2009.12.046

[10] Pump A , Di Biase L , Price J , et al. Efficacy of catheter ablation in nonparoxysmal atrial fibrillation patients with severe enlarged left atrium and its impact on left atrial structural remodeling. J Cardiovasc Electrophysiol. 2013;24(11):1224‐1231. 24020717 10.1111/jce.12253

[11] Kim JS , Im SI , Shin SY , et al. Changes in left atrial transport function in patients who maintained sinus rhythm after successful radiofrequency catheter ablation for atrial fibrillation: a 1‐year follow‐up multislice computed tomography study. J Cardiovasc Electrophysiol. 2017;28(2):167‐176. 27859888 10.1111/jce.13128

[12] Canpolat U , Aytemir K , Ozer N , Oto A . The impact of cryoballoon‐based catheter ablation on left atrial structural and potential electrical remodeling in patients with paroxysmal atrial fibrillation. J Interv Card Electrophysiol. 2015;44(2):131‐139. 26238480 10.1007/s10840-015-0041-1

[13] Shrestha S , Chen O , Greene M , John JJ , Greenberg Y , Yang F . Change in P wave morphology after convergent atrial fibrillation ablation. Indian Pacing Electrophysiol J. 2016;16(1):3‐7. 27485559 10.1016/j.ipej.2016.02.010 PMC4936606

[14] Maan A , Mansour M , Ruskin JN , Heist EK . Impact of catheter ablation on P‐wave parameters on 12‐lead electrocardiogram in patients with atrial fibrillation. J Electrocardiol. 2014;47(5):725‐733. 24850319 10.1016/j.jelectrocard.2014.04.010

[15] Fujimoto Y , Yodogawa K , Takahashi K , et al. Noninvasive evaluation of reverse atrial remodeling after catheter ablation of atrial fibrillation by P wave dispersion. Heart Vessel. 2017;32(11):1375‐1381. 10.1007/s00380-017-1008-1 28631077

[16] Lazzerini PE , Laghi‐Pasini F , Acampa M , et al. Systemic inflammation rapidly induces reversible atrial electrical Remodeling: the role of Interleukin‐6‐mediated changes in Connexin expression. J Am Heart Assoc. 2019;8(16):e011006. 31423933 10.1161/JAHA.118.011006 PMC6759884

[17] Okutucu S , Aytemir K . Oto a. P‐wave dispersion: what we know till now? JRSM Cardiovasc Dis. 2016;5:2048004016639443. 27081484 10.1177/2048004016639443 PMC4814939

[18] Kizilirmak F , Demir GG , Gokdeniz T , et al. Changes in electrocardiographic P wave parameters after cryoballoon ablation and their association with atrial fibrillation recurrence. Ann Noninvasive Electrocardiol. 2016;21(6):580‐587. 27018476 10.1111/anec.12364 PMC6931592

[19] Miyazaki S , Kuwahara T , Kobori A , et al. Preprocedural predictors of atrial fibrillation recurrence following pulmonary vein antrum isolation in patients with paroxysmal atrial fibrillation: long‐term follow‐up results. J Cardiovasc Electrophysiol. 2011;22(6):621‐625. 21235666 10.1111/j.1540-8167.2010.01984.x

[20] Njoku A , Kannabhiran M , Arora R , et al. Left atrial volume predicts atrial fibrillation recurrence after radiofrequency ablation: a meta‐analysis. Europace. 2018;20(1):33‐42. 28444307 10.1093/europace/eux013

[21] Thomas L , Abhayaratna WP . Left atrial reverse Remodeling: mechanisms, evaluation and clinical significance. JACC Cardiovasc Imaging. 2017;10(1):65‐77. 28057220 10.1016/j.jcmg.2016.11.003

[22] Chavez‐Gonzalez E , Donoiu I . Utility of P‐wave dispersion in the prediction of atrial fibrillation. Curr Health Sci J. 2017;43(1):5‐11. 30595848 10.12865/CHSJ.43.01.01 PMC6286725

[23] Kurose J , Kiuchi K , Fukuzawa K , et al. The lesion characteristics assessed by LGE‐MRI after the cryoballoon ablation and conventional radiofrequency ablation. J Arrhythm. 2018;34(2):158‐166. 29657591 10.1002/joa3.12025 PMC5891401

[24] Wieczorek M , Tajtaraghi S , Sassani K , Hoeltgen R . Incidence of early pulmonary vein reconnections using different energy sources for pulmonary vein isolation: multielectrode phased radiofrequency vs second‐generation cryoballoon. J Cardiovasc Electrophysiol. 2019;30(9):1428‐1435. 31111548 10.1111/jce.13991

[25] Andrade JG , Dubuc M , Guerra PG , et al. The biophysics and biomechanics of cryoballoon ablation. Pacing Clin Electrophysiol: PACE. 2012;35(9):1162‐1168. 22671922 10.1111/j.1540-8159.2012.03436.x

[26] Ejima K , Arai K , Suzuki T , et al. Long‐term outcome and preprocedural predictors of atrial tachyarrhythmia recurrence following pulmonary vein antrum isolation‐based catheter ablation (RFA)in patients with non‐paroxysmal atrial fibrillation. J Cardiol. 2014;64(1):57‐63. 24373867 10.1016/j.jjcc.2013.11.010

[27] Marrouche NF , Wilber D , Hindricks G , et al. Association of atrial tissue fibrosis identified by delayed enhancement MRI and atrial fibrillation catheter ablation: the DECAAF study. JAMA. 2014;311(5):498‐506. 24496537 10.1001/jama.2014.3

